# Comparing patient reported abdominal pain between patients treated with oxaliplatin-based pressurized intraperitoneal aerosol chemotherapy (PIPAC-OX) and primary colorectal cancer surgery

**DOI:** 10.1038/s41598-023-47510-0

**Published:** 2023-11-22

**Authors:** Vincent C. J. van de Vlasakker, Robin J. Lurvink, Emma C. Wassenaar, Paulien Rauwerdink, Checca Bakkers, Koen P. Rovers, Cynthia S. Bonhof, Jacobus W. A. Burger, Marinus J. Wiezer, Djamila Boerma, Simon W. Nienhuijs, Floortje Mols, Ignace H. J. T. de Hingh

**Affiliations:** 1 Department of Surgery, Catharina Hospital, Catharina Cancer Institute, PO Box 1350, 5602 ZA Eindhoven, The Netherlands; 2 Department of Research, Netherlands Cancer Registry, IKNL, Utrecht, The Netherlands; 3 Department of Surgery, St. Antonius Hospital, Nieuwegein, The Netherlands; 4 Department of Medical and Clinical Psychology, CoRPS – Centre of Research on Psychological Disorders and Somatic Diseases, Tilburg University, Tilburg, The Netherlands; 5 GROW – School for Oncology and Developmental Biology, Maastricht University, Maastricht, The Netherlands

**Keywords:** Cancer, Surgical oncology, Cancer, Diseases, Medical research, Oncology

## Abstract

Oxaliplatin-based pressurized intraperitoneal aerosol chemotherapy (PIPAC-OX) is an emerging palliative treatment for patients with unresectable colorectal peritoneal metastases. Previously, our study group reported that patients experienced abdominal pain for several weeks after PIPAC-OX. However, it is unknown how this compares to abdominal pain after regular colorectal cancer surgery. To provide some perspective, this study compared the presence of abdominal pain after PIPAC-OX to the presence of abdominal pain after primary tumor surgery. Patient reported abdominal pain scores (EORTC QLQ-CR-29), from two prospective, Dutch cohorts were used in this study. Scores ranged from 0 to 100, a higher score represents more abdominal pain. Abdominal pain at baseline and at four weeks after treatment were compared between the two groups. Twenty patients who underwent PIPAC-OX and 322 patients who underwent primary tumor surgery were included in the analysis. At baseline, there were no differences in abdominal pain between both groups (mean 20 vs. 18, respectively; *p* = 0.688). Four weeks after treatment, abdominal pain was significantly worse in the PIPAC group (39 vs 15, respectively; *p* < 0.001; Cohen’s d = 0.99). The differential effect over time for abdominal pain differed significantly between both groups (mean difference: 19 vs − 3, respectively; *p* = 0.004; Cohen’s d = 0.88). PIPAC-OX resulted in significantly worse postoperative abdominal pain than primary tumor surgery. These results can be used for patient counseling and stress the need for adequate analgesia during and after PIPAC-OX. Further research is required to prevent or reduce abdominal pain after PIPAC-OX.

*Trial registration* CRC-PIPAC: Clinicaltrails.gov NCT03246321 (01-10-2017)

## Introduction

Oxaliplatin-based pressurized intraperitoneal aerosol chemotherapy (PIPAC-OX) is a new palliative treatment option for patients with unresectable colorectal peritoneal metastases (CPM)^1–4^. Given the lack of prospective studies^5^, the CRC-PIPAC study was conducted to prospectively investigate the safety, feasibility, preliminary efficacy of repetitive PIPAC-OX monotherapy in twenty patients with unresectable CPM^6,7^. A secondary aim was to explore patient-reported outcomes (PROs) during trial treatment^8^. It was observed that several PROs were affected, but ultimately all PROs remained unaffected or recovered over time except one PRO, abdominal pain. While abdominal pain did not worsen cumulatively, it worsened significantly after each PIPAC procedure^8^. The presence of abdominal pain after treatment with PIPAC-OX was also reported by other researchers^9^.

Given the novelty of PIPAC-OX, it is unknown how the presence of abdominal pain after PIPAC-OX compares to the occurrence of abdominal pain after other surgical interventions within colorectal cancer (CRC) treatment, such as primary tumor surgery (PTS). Therefore, the aim of this study was to compare the PRO *abdominal pain* in patients treated with PIPAC-OX for unresectable CPM to CRC patients undergoing PTS and the results of this study will provide more insight into the burden of PIPAC-OX.

## Methods

### Study setting and population

This study compared prospectively collected PROs from patients who were enrolled in the CRC-PIPAC study (NCT03246321 [01-10-2017]) to patients who were enrolled in the PROCORE study (NL51119.060.14 [01-01-2016])^6,10^. The CRC-PIPAC study was conducted in two Dutch hospitals and the PROCORE study was conducted in four Dutch hospitals. Both studies were approved by a central medical ethics committee (Medical Research Ethics Committees United [MEC-U]) and institutional review boards of all participating study centers (the review boards of the Elisabeth-TweeSteden hospital, Catharina hospital, Elkerliek hospital, and Máxima Medical Centre for the PROCORE study and the review boards of the St. Antonius hospital and Catharina hospital for the CRC-PIPAC study). Informed consent was obtained from all participating patients and the research was performed in accordance with the Declaration of Helsinki.

The CRC-PIPAC study was a single-arm phase 2 clinical trial that prospectively enrolled twenty patients with isolated unresectable CPM between October 2017 and September 2018. Patients underwent PIPAC-OX (92 mg/m^2^) under general anesthesia with a simultaneous bolus of intravenous 5-fluorouracil (400 mg/m^2^) and leucovorin (20 mg/m^2^). Oxaliplatin was prepared in a total volume of 150 mL dextrose solution and injected through the nebulizer (CapnoPen, Capnomed GmbH, Villingendorf, Germany) in 5 min, after which the Ultravision generator (Ultravision, Alesi Surgical, Cardiff, UK) administered electrostatic precipitation to the aerosol. The electrostatic field and the capnoperitoneum were maintained for 25 min. PIPAC-OX procedures were repeated every six weeks, until disease progression, unacceptable toxicity, physicians decision-, or patient’s request to discontinue. All patients who underwent at least one PIPAC-OX were included and only results from the first PIPAC-OX were used in the comparative analyses.

The PROCORE study was a prospective population-based study that enrolled patients with all stages of colorectal cancer between 1 January 2016 and 31 December 2018. The main goal of the PROCORE study was to collect PROs in a large population-based cohort of colorectal cancer patients who were treated according to the Dutch guidelines (i.e. no trial treatment was given as part of the PROCORE study). All patients diagnosed with stage 2–4 colorectal cancer between 1 January 2016 and 31 December 2018 who underwent PTS were included in the comparative analyses.

Post-operative management for patients of both groups was in accordance to early recovery after surgery (ERAS) guidelines, meaning that analgesia protocols prescribed opioids only if necessary and only short-term.

### Abdominal pain assessment

Patients in the CRC-PIPAC study were asked to complete PRO questionnaires at baseline and at one and four weeks after PIPAC-OX. Patients in the PROCORE study were asked to complete PRO questionnaires at baseline and four weeks after PTS. In both studies, the European Organization for Research and Treatment of Cancer Quality of Life Questionnaire (EORTC QLQ) CR29 questionnaire was used^11,12^. Only one PRO was included in this study: *abdominal pain* (EORTC QLQ-CR29). PRO scores were calculated according to the corresponding manual^13^. Scores range from 0 to 100, a higher score represents more abdominal pain.

### Statistical analyses

Categorical baseline characteristics were presented as n (%) and compared with the Chi-square test. PRO scores of both groups were presented as mean with standard deviation. Differential effects in abdominal pain scores over time and scores at both time points (i.e. baseline and four weeks after surgery / first PIPAC procedure) were compared between PTS-patients and PIPAC-OX-patients using linear mixed modeling (LMM). For LMM maximum likelihood estimation and an unstructured covariance matrix were used. The covariance matrix consisted of a two-level structure where the two repeated time points (i.e. baseline and 4 weeks post-operative) represented the lower levels and individual patients represented the higher level. Parameters known to potentially affect pain (e.g. age, sex, and tumor location) were included in the model as covariates and subsequently removed if they did not improve the model. Statistical significance was set at *p* < 0.01 to adjust for multiple testing. The Cohen’s D (CD) was calculated to determine the clinical relevance (i.e. > 0.500). IBM SPSS Statistics (version 25.0 Armonk, NY, United States) was used for all analyses.

## Results

The study population comprised 342 patients: 322 underwent PTS and 20 underwent PIPAC-OX.

The baseline characteristics are provided in Table 1. Patients in the PIPAC-OX group more often had a primary tumor located in the right colon and had more often received systemic treatment prior to enrollment than patients in the PTS group. All patients in the PIPAC-OX group had stage IV disease, whereas most patients in the PTS group had stage II or II disease.

**Table 1:** Baseline characteristics.

	PTS (n = 322)	PIPAC-OX (n = 20)	*P* value
Sex			0.850
Female	122 (38%)	8 (40%)	
Age			0.211
< 50 years	14 (4%)	2 (10%)	
50–70 years	172 (53%)	13 (65%)	
> 70 years	136 (42%)	5 (25%)	
Primary tumor location			**0.001**
Right colon	107 (33%)	14 (70%)	
Left colon	115 (36%)	6 (30%)	
Rectum	100 (31%)	0 (0%)	
Previous systemic treatment			** < 0.001**
No	278 (86%)	8 (40%)	
Yes	44 (14%)	12 (60%)	
Stage at enrollment			** < 0.001**
2	133 (41%)	0 (0%)	
3	170 (53%)	0 (0%)	
4	19 (6%)	20 (100%)	

*PIPAC-OX* Pressurized Intraperitoneal Aerosol Chemotherapy with oxaliplatin,* PTS* primary tumor surgery.

Significant values are in bold.

Among those in the PTS group, 104 (33%) patients underwent a right hemicolectomy or resection of the transverse colon; 109 (34%) patients underwent a left hemicolectomy or resection of the sigmoid colon; 99 (31%) patients underwent a low anterior or abdominoperineal resection; and 6 (2%) patients underwent a subtotal colectomy.

### Abdominal pain

**Figure 1:**
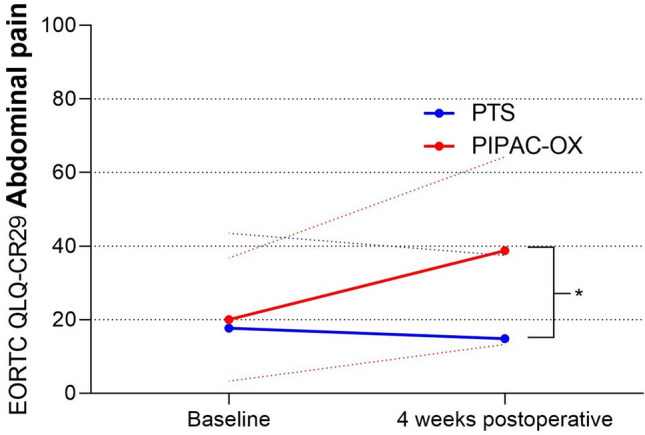
Symptom scores for abdominal pain. Blue and red lines represent mean scores; dotted blue and pink lines represent standard deviations; Marking with an asterisk (*) represents a statistically significant and clinically relevant difference; PIPAC-OX, oxaliplatin-based pressurized intraperitoneal aerosol chemotherapy; PTS, primary tumor surgery.

At baseline, abdominal pain did not differ significantly between both groups (mean score: 20 vs. 18, respectively; *p* = *0.688*). From baseline to four weeks postoperative, abdominal pain did not worsen in the PTS group (mean score: 18 vs. 15, respectively; *p* = 0.163), but worsened significantly in the PIPAC-OX group (mean score: 20 vs. 39, respectively; *p* = *0.004, CD* = *0.88*). This differential effect was significantly different between both patient groups (mean difference: 19 vs − 3; *p* < *0.001*) (Table 2, Fig. 1).

**Table 2:** EORTC QLQ-CR29 abdominal pain scores among colorectal cancer patients undergoing PIPAC-OX or PTS.

	Baseline				4 weeks postoperative			
	PIPAC-OX	PTS	*p* value	Cohens D	PIPAC-OX	PTS	*p* value	Cohens D
Abdominal pain	20 ± 17	18 ± 26	0.688	–	39 ± 25	15 ± 23	** < 0.001**	**0.998**

All values are mean ± standard deviation; *PIPAC-OX* Pressurized Intraperitoneal Aerosol Chemotherapy with oxaliplatin,* PTS* primary tumor surgery. All scores range from 0 to 100. A higher score on the abdominal pain scale represents more abdominal pain.

Significant and clinically relevant values are in bold.

None of the covariates (Sex, age, and tumor location) that were tested significantly improved the model and were therefore omitted.

## Discussion

This study aimed to compare *abdominal pain* of patients with colorectal peritoneal metastases treated with PIPAC-OX to patients with colorectal cancer treated with primary tumor surgery. At four weeks postoperative, patients treated with PIPAC-OX experienced significantly more abdominal pain than patients treated with PTS. This is a counterintuitive finding, since no visceral resections are performed during PIPAC-OX, whereas visceral resection are regularly performed during PTS.

One of the major proclaimed benefits of PIPAC-OX is the limited effect of this treatment on quality of life, which would be favorable in the palliative setting. However, the current study suggests that patients undergoing PIPAC-OX experience significantly more abdominal pain than patients undergoing PTS. Patients undergoing PIPAC-OX have a more advanced cancer stage than PTS patients, therefore PIPAC-OX patients are more at risk for oncological pain, especially since no resections are performed in this patient group^14^. However, the previously published CRC-PIPAC study showed an increase in abdominal pain one week after each procedure followed by a relative decrease in abdominal pain three weeks later, suggesting that the increase in abdominal pain is at least in part due to PIPAC-OX^8^. Given the repetitive intent of treatment with PIPAC-OX, these palliative patients are repetitively exposed to abdominal pain. This should be considered by both treating physicians and patients before starting treatment and it stresses the need for adequate analgesic protocols.

Other studies investigating PIPAC in a palliative setting have also suggested that treatment with PIPAC-OX is less well-tolerated than initially thought, as it may result in chemical peritonitis or even peritoneal sclerosis, leading to abdominal pain^8,15^. The effect of PIPAC on abdominal pain may be drug dependent, as two studies reported a greater inflammatory response^16^ and a greater morphine demand^17^ after PIPAC-OX than after PIPAC with cisplatin/doxorubicin. Furthermore, although a dose-dependent effect of PIPAC-OX on abdominal pain was not reported in two dose-escalating studies in the palliative setting^18,19^, a third study investigating PIPAC-OX in the adjuvant setting (i.e. high-risk colorectal cancer patients) observed severe abdominal pain in the majority of patients treated with PIPAC-OX, who required either dose-reduction of intraperitoneal oxaliplatin or discontinuation of adjuvant PIPAC-OX^9^. However, the different setting of this third study (adjuvant instead of palliative) may explain the observed dose-dependent effect on abdominal pain, since patients in the palliative setting might be more willing to accept (severe) adverse events from a last-resort treatment.

While seven other studies also presented PROs during PIPAC with various drugs for various primary tumors among which PIPAC-OX for CPM^19–25^, three did not provide PRO results on abdominal pain^20–22^. Of the other four, one reported a transitory increase in pain^23^ whereas the other three reported that pain remained stable during treatment with PIPAC^24–26^. However, these studies did not provide separate PRO results for PIPAC-OX in patients with CPM^23–25^ nor for PIPAC-OX monotherapy^26^, which impedes the interpretation of these findings. In addition, a recent study reported increased pain levels after repetitive PIPAC treatment, as measured through patient reported outcomes^27^. While some of these studies report increased pain levels, they do not provide context to these increased pain levels. The results of the present study show that the severity of abdominal pain after PIPAC is higher than that after conventional surgery, thereby providing important insights into the pain burden caused by PIPAC-OX.

Although this is the first study to compare abdominal pain in patients with CPM who were treated with PIPAC-OX to patients with colorectal cancer who underwent PTS, there are some limitations to this study. First, the PROCORE study population resembles a population-based study, since all patients with colorectal cancer were allowed to participate. In contrast, the PIPAC-OX study population consists of a highly selected group of palliative patients who required to be in an adequate clinical condition to participate in a clinical trial, which might have affected baseline quality of life. Second, the timing of only two PRO measurements matched between the two studies (i.e. baseline and four weeks after surgery). If the timing of more PRO measurements had matched, this could have provided more insight in short-term (e.g. one week after surgery) and longer-term (e.g. several months after surgery) differences in PROs between the two groups. Given the poor prognosis of patients in the PIPAC-OX group, long-term PRO measurements were not available in this group. While these limitations may hinder the generalizability of the results to some extent, they do provide important information on patient reported pain after PIPAC-OX treatment, even if it only concerns the short-term, non-repetitive treatment situation. A third limitation concerns the operation-technique. Due to the registration in the PROCORE study, no information could be obtained regarding operation technique (e.g. open vs. laparoscopic). It is to be expected that an open technique would result in more post-operative pain, as the surgical trauma is larger. This might have increased the reported abdominal pain scores in the PTS group and might have attenuated the differential effect found in this study. A comparative study between laparoscopic PTS and PIPAC might thus result in an even bigger difference in abdominal pain as in the Netherlands approximately 32% of patients with CRC underwent open surgery, while 68% underwent laparoscopic surgery in 2015^28^. However, the present finding simultaneously signifies the difference in abdominal pain, which was greater after PIPAC despite the PTS group more frequently undergoing open surgery.

## Conclusions

Patients with colorectal peritoneal metastases who were treated with PIPAC-OX reported significantly more abdominal pain at four weeks postoperative than patients with colorectal cancer who underwent PTS. Although this effect might in part be caused by a more advanced tumor stage, it nevertheless indicates that physicians should not underestimate the longevity and severity of abdominal pain after PIPAC-OX. Physicians should counsel patients accordingly and should consider analgesic measures to reduce these symptoms, especially since PIPAC-OX is mainly applied in the palliative setting and is often repeated several times. Future studies should focus on the development of analgesic protocols aiming to reduce abdominal pain during treatment with PIPAC-OX.

## Data Availability

Data that were analysed in this study will be made readily available by the corresponding author upon reasonable written request.

## References

[1] Solass W, Hetzel A, Nadiradze G (2012). Description of a novel approach for intraperitoneal drug delivery and the related device. Surg. Endosc..

[2] Solass W, Herbette A, Schwarz T (2012). Therapeutic approach of human peritoneal carcinomatosis with Dbait in combination with capnoperitoneum: Proof of concept. Surg. Endosc..

[3] Solass W, Kerb R, Mürdter T (2014). Intraperitoneal chemotherapy of peritoneal carcinomatosis using pressurized aerosol as an alternative to liquid solution: First evidence for efficacy. Ann. Surg. Oncol..

[4] Blanco A, Giger-Pabst U, Solass W (2013). Renal and hepatic toxicities after pressurized intraperitoneal aerosol chemotherapy (PIPAC). Ann. Surg. Oncol..

[5] Lurvink, R. J., Rovers, K. P., Nienhuijs, S. W., Creemers, G. J., Burger, J. W. A., de Hingh, I. H. J. T. Pressurized intraperitoneal aerosol chemotherapy with oxaliplatin (PIPAC-OX) in patients with colorectal peritoneal metastases—A systematic review. *J Gastrointest Oncol.* Apr; **12**(Suppl 1): S242–S25 (2020). 10.21037/jgo-20-257 PMC8100707 33968441

[6] Rovers KP, Lurvink RJ, Wassenaar ECE (2019). Repetitive electrostatic pressurised intraperitoneal aerosol chemotherapy (ePIPAC) with oxaliplatin as a palliative monotherapy for isolated unresectable colorectal peritoneal metastases: Protocol of a Dutch, multicentre, open-label, single-arm, phase II study (CRC-PIPAC). BMJ Open..

[7] Rovers KP, Wassenaar ECE, Lurvink RJ (2021). Pressurized intraperitoneal aerosol chemtoherapy (oxaliplatin) for unresectable colorectal peritoneal metastases: A multicenter, single-arm, phase II trial (CRC-PIPAC). Ann. Surg. Oncol..

[8] Lurvink RJ, Rovers KP, Wassenaar ECE (2021). Patient-reported outcomes during repetitive oxaliplatin-based pressurized intraperitoneal aerosol chemotherapy for isolated unresectable colorectal peritoneal metastases in a multicenter, single-arm, phase 2 trial (CRC-PIPAC). Surg. Endosc..

[9] Graversen M, Detlefsen S, Pfeiffer P, Mortensen MB (2021). Local peritoneal toxicity from adjuvant pressurized intraperitoneal aerosol chemotherapy with oxaliplatin in high-risk patients with colonic cancer. BJS..

[10] Bonhof CS, van de Poll-Franse LV, Wasowicz DK, Beerepoot LV, Vreugdenhil G, Mols F (2020). The course of peripheral neuropathy and its association with health-related quality of life among colorectal cancer patients. J. Cancer Surviv..

[11] Aaronson NK, Ahmedzai S, Bergman B (1993). The European organization for research and treatment of cancer QLQ-C30: A quality-of-life instrument for use in international clinical trials in oncology. J. Natl. Cancer Inst..

[12] Stiggelbout AM, Kunneman M, Baas-Thijssen MC (2016). The EORTC QLQ-CR29 quality of life questionnaire for colorectal cancer: validation of the Dutch version. Qual. Life Res..

[13] Whistance RN, Conroy T, Chie W (2009). Clinical and psychometric validation of the EORTC QLQ-CR29 questionnaire module to assess health-related quality of life. Eur. J. Cancer..

[14] Van den Beuken-van Everdingen M, De Rijke J, Kessels A, Schouten H, Van Kleef M, Patijn J (2007). Prevalence of pain in patients with cancer: a systematic review of the past 40 years. Ann. Oncol..

[15] Graversen M, Detlefsen S, Pfeiffer P, Lundell L, Mortensen MB (2018). Severe peritoneal sclerosis after repeated pressurized intraperitoneal aerosol chemotherapy with oxaliplatin (PIPAC OX): Report of two cases and literature survey. Clin. Exp. Metastasis.

[16] Teixeira Farinha H, Grass F, Labga I, Pache B, Demartines N, Hübner M (2018). Inflammatory response and toxicity after pressurized intraperitoneal aerosol chemotherapy. J. Cancer.

[17] Graversen M, Lundell L, Fristrup C, Pfeiffer P, Mortensen MB (2018). Pressurized intraperitoneal aerosol chemotherapy (PIPAC) as an outpatient procedure. Pleura Peritoneum.

[18] Dumont F, Passot C, Raoul JL (2020). A phase I dose-escalation study of oxaliplatin delivered via a laparoscopic approach using pressurised intraperitoneal aerosol chemotherapy for advanced peritoneal metastases of gastrointestinal tract cancers. Eur. J. Cancer.

[19] Kim G, Tan HL, Sundar R (2021). PIPAC-OX: A phase I study of oxaliplatin-based pressurized intraperitoneal aerosol chemotherapy in patients with peritoneal metastases. Clin. Cancer Res..

[20] Robella M, Vaira M, de Simone M (2016). Safety and feasibility of pressurized intraperitoneal aerosol chemotherapy (PIPAC) associated with systemic chemotherapy: An innovative approach to treat peritoneal carcinomatosis. World J. Surg. Oncol..

[21] Graversen M, Detlefsen S, Bjerregaard JK, Fristrup CW, Pfeiffer P, Mortensen MB (2018). Prospective, single-center implementation and response evaluation of pressurized intraperitoneal aerosol chemotherapy (PIPAC) for peritoneal metastasis. Ther. Adv. Med. Oncol..

[22] Taibi, A., Teixeira Farinha, H., Durand Fontanier, S., Sayedalamin, Z., Hübner, M. & Sgarbura, O. Pressurized intraperitoneal aerosol chemotherapy enhanced by electrostatic precipitation (ePIPAC) for patients with peritoneal metastases.* Ann. Surg. Oncol*. (2020) 10.1245/s10434-020-09332-6 33216263

[23] Odendahl K, Solass W, Demtröder C (2015). Quality of life of patients with end-stage peritoneal metastasis treated with pressurized intraperitoneal aerosol chemotherapy (PIPAC). Eur. J. Surg. Oncol..

[24] Teixeira Farinha H, Grass F, Kefleyesus A (2017). Impact of pressurized intraperitoneal aerosol chemotherapy on quality of life and symptoms in patients with peritoneal carcinomatosis: A retrospective cohort study. Gastroenterol. Res. Pract..

[25] de Simone M, Vaira M, Argenziano M (2020). Pressurized intraperitoneal aerosol chemotherapy (PIPAC) with oxaliplatin, cisplatin, and doxorubicin in patients with peritoneal carcinomatosis: an open-label, single-arm, phase II clinical trial. Biomedicines.

[26] Tabchouri N, Buggisch J, Demtroder CR (2021). Pressurized intraperitoneal aerosol chemotherapy for colorectal peritoneal metastases. Ann. Surg. Oncol..

[27] Graversen M, Detlefsen S, Ainsworth AP, Fristrup CW, Knudsen AO, Pfeiffer P (2023). Treatment of peritoneal metastasis with pressurized intraperitoneal aerosol chemotherapy: Results from the prospective PIPAC-OPC2 study. Ann. Surg. Oncol..

[28] Lurvink R, Bakkers C, Rijken A, Van Erning F, Nienhuijs S, Burger J (2021). Increase in the incidence of synchronous and metachronous peritoneal metastases in patients with colorectal cancer: A nationwide study. Eur. J. Surg. Oncol..

